# The Foster-Brothers

**Published:** 1881-03

**Authors:** 


### THE FOSTER-BROTHERS.

CONTINUED.

HER eyes lighted on Brydges for an instant as she played the iast lively bars.

"I hope you will remain some time," she said. "I have heard Mr. Marshall say so much of you that I have been quite anxious to know you."

Brydges hardly knew whether to be pleased or vexed. This lovely, intrepid, self-possessed girl, treating him with this utterly unconventional frankness, was not at all flattering to his *amour-propre.* He jumped to the hasty conclusion that she must be *fiancée* to Marshall. He felt half inclined to hate them both. He hated himself worse for feeling embarrassed by the steady glance of the soft brown eyes.

"Yes—that is, not long," he stammered ; then added with unnecessary emphasis, "I am going to-morrow."

Marshall laughed and said, "Mimi, he will spend a week or two with us. Your music shall soothe his savage breast till we get tired of him and send him on his way, a sadder and a better man."

They rose to go. Miss Des Ponts rang, and a silver-haired negro answered.

"Take these gentlemen to the gate, Darby ;" but glancing out of the window, she exclaimed, "No! *violà*, papa! I will go myself."

As they sculled over the garden Marshall said, "Des Ponts translates Brydges."

"Not oversets, I hope, as Father Krakwity would say," she answered, laughing.

Mr. Des Ponts stood at the gate. There was a hurried introduction and word of greeting.

"My mother expects you every day, and hereafter we wait dinner for you," said Marshall.

Des Ponts looked troubled and anxious.

"I fear we *must* very soon claim your hospitality. This rise looks serious. The Des Moines is full of back-water for miles. The 'oldest inhabitants' are talking like screech-owls this afternoon."

"Never mind, *mon petit* papa. Here's a sigh for those that love us, and a smile for those that hate, and—and—before it gets above us, perhaps it may abate ;" and father and daughter sculled to their beleaguered mansion.

As Marshall and Brydges walked to the ferry they saw evident signs of consternation among the towns-people. Those who lived in two-story houses were engaged in emptying their ground-floors, while the groundlings were begging room "under the shingles" from their more fortunate neighbors. Still, some *esprits forts* were walking calmly about deriding and pooh-poohing, and demonstrating by all the almanacs known that this "was not a high-water year."

That evening the young gentlemen were smoking on the veranda in the dim, confidential starlight. Brydges said, apropos of nothing: "Cade, I congratulate you. Miss Des Ponts is an excessively pretty girl."

"My dear Clarence, you have more taste than sagacity. I have no property whatever in Mimi Des Ponts' unquestionable beauty."

"Why not?" rejoined Brydges, in a somewhat querulous tone. "You don't mean that there are more of that style of girls in the neighborhood; and whom, besides you, would *she* look at hereaway?"

"It is very sweet of you, gentle stranger, to say such things of us. Mimi and I love each other too well to be lovers, I suppose. I never had any sister but her; nor she ever a brother but me. We made mud-pies together, and fought over the first strawberries of the season. But I have never thought of availing myself of my evident advantages. I have magnanimously waited for some handsome pilgrim with blue eyes to come, and, if worthy and enterprising, to win her."

"*Elle vaut bien la peine.*"

"*You* have the requisite Gothic complexion; you will have idleness and juxtaposition in your favor in a day or two. The great river is working valiantly for you to-night."

"What a superb picture of quiet power!" said Brydges. "There it flows, pouring out over the level bottoms the flood of ten thousand thunder-storms, annihilating farms, fields, and villages, and not the murmur of a ripple comes up to us in this deep silence. It was a true artistic thought of the old religions that made gods of the rivers."

"Yes. I think even Carlyle would respect the Mississippi—so much work with so little talk."

From the window of his chamber that night Clarence Brydges looked out once more upon the vast and broadening sheet of water, and the twinkling lights of the village by the shore. One, he fancied, without any reason except its brightness, was lighting Marie

Des Ponts to rest. He gazed musingly at this light till it suddenly disappeared.

"Good-night, and happy dreams," he murmured; then added, "Well, I have given that dark-eyed Missourian enough of my thoughts to-night," and went on thinking of nothing else till he fell asleep.

In the morning, as he came out upon the veranda, he saw the Colonel gazing intently at something in the river. "Cade, my son, get my field-glass. Good-morning, Mr. Brydges, I hope you had pleasant dreams your first night at Fort Johnstone. You know they are to come true, according to our received traditions."

Cade handed him the glass. He glanced at the object that had puzzled him, and laughed—a hearty, strutting, crowing sort of laugh, and handed the glass to Brydges. "There, I don't believe even so blasé a veteran as yourself ever saw a sight like that before."

It was a chicken-coop floating down the river, its hapless inmates roosting on the roof with an air of draggled and desperate resignation. It was a slight but most significant specimen of the night's work.

"Look across the river, Mr. Brydges. The ponds of yesterday are lakes and bays. Behind the town the prairie is one vast sheet of water to the bluffs. Below us the Illinois shore is invaded ; the bottom will be flooded to-morrow."

"We shall have the Des Ponts to dinner, doubtless."

"Yes; and then, Mr. Brydges, look out for your heart, if you carry any such light baggage."

The theme was one on which the old gentleman was always eloquent. He began his usual rhapsody, but was soon interrupted by a summons to breakfast.

"Who is Mr. Des Ponts?" asked Brydges, when they were seated at table.

"Lawyer by profession, gentleman by practice," said Cade.

"The richest man in Thebes, and the best bred man in Missouri," said Mrs. Marshall.

"He is a French creole," said the Colonel, "who has the good taste to speak English without lisping. He has good books and good wine, and he buys both himself. The most curious thing about him is that every body owes him money and nobody hates him."

"But," said Clarence, "why does this phenix of Missourians live in the Theban waste?"

"Ah, that is his most amiable point," said Mrs. Marshall. "He is bound by a promise to the late Madame Des Ponts. She was an enthusiastic Southern woman, who thought a free State the abomination of desolation; even wrote a florid pamphlet called the 'Curse of Canaan;' she said she did not see how one could be a Christian and not own slaves, when their means permitted; and I believe honestly doubted whether negroes had souls. She has often said to me that she wished it could be shown that a certain famous text should read in the original, 'Suffer little white children to come unto me.'"

Every one laughed except Mr. Brydges.

"I always thought," the jolly old lady went on, "that Des Ponts recognized as clearly as any one the absurdity of Madame's opinions. But he never disputed with her, and often, when she was hard pressed in a discussion, he would come to her rescue with some brilliant paradox that left one in doubt which side he was really on. She never doubted, I am sure. I never saw a husband so worshipped by his wife. Though one of the proudest of the Shelbys, she delighted in displaying her entire subjection to him. I believe she would have polished his boots if he had permitted it."

"*O! si sic omnes*," said the Colonel, and the young men groaned in unison.

Mrs. Marshall continued, scorning the interruption: "She never lost her early infatuation for Des Ponts. The very year she died she and I were in the drawing-room, and Mr. Marshall and he were on the veranda. She looked at him some time in rapt contemplation, and said at last: 'Who could look at that noble form and god-like brow and then think without disgust of Jefferson's clap-trap of the equality of men?'"

"She would have preferred," said Cade, "the dictum of our Pomp—'One man is as good as anuddah, an' a heap bettah.'"

"When her last illness came, she seemed to regret nothing but leaving Des Ponts. She would not be pacified till he swore—most reluctantly, and after a terrible scene—that he would never take Marie to a Northern State. For she said she hoped still to be with them in spirit, and she could not follow them into Yankee barbarism. So, ever since, poor Des Ponts has lived in that hideous swamp—the Despontine Marshes, as Cade says."

"But why not go South?" said Brydges.

"I imagine he prefers, while keeping his vow faithfully, to live in this extreme corner of slave territory, in sight of free sky and soil."

Brydges bit his lip; and Mrs. Marshall, remembering too late from what latitude he came, talked of pleasant trifles, and put too much sugar in his second cup of coffee by way of apology.

About noon the wagoner, Chris, having reclaimed his cart from its funereal functions, drove up to the back-door, and leaping down, shouldered a vast Saratoga trunk, with which he marched into the house.

"Vere I packs him? Die schoene Fräulein is comin' bimeby a'ready mit 'm Herr Vater. Mein Gott! Die is wunderschoen," he said, grotesquely kissing his stubby finger-ends.

By the time the luggage was bestowed the exiles were at the door.

The Colonel met them with his hearty, old-fashioned courtesy:

"La Rochefoucauld was right. There *is* something in the misfortunes of our best friends that does not altogether displease us."

He shook hands with Des Ponts and kissed the neat glove of Marie. She nodded smilingly to the young men, and entered the house with Mrs. Marshall.

"I should have come yesterday," said Des Ponts, "had it not been for the indomitable Shelby pluck of Mimi. We moved the furniture to the second floor in the afternoon; but she still insisted that the river would fall, and so we drank tea in the dismantled parlor, and sat by the fire until the water poured over the floor and flooded the hearth. "What am I to do with my feet?' she coolly inquired. "Would it be quite lady-like to put them on the mantle-piece?" I took her in my arms and waded to the stairs, and carried her up to bed. This morning she got into the skiff from her chamber-window by a rope-ladder—and ever since she calls me Romeo!"

While he was speaking Brydges observed him more closely than he had previously done. He was certainly a strikingly handsome man; a clear, dark skin; black eyes under straight brows; a square forehead and resolute jaw; the mouth almost concealed by a grizzled mustache, a feature not then so common as now; the whole face framed with glossy and luxuriant black curls. There was a strong general resemblance to his daughter; yet they were curiously unlike. The fine animal beauty of his face was in hers lit up and spiritualized by the glancing light of a vivid intelligence. Seeing them together you would think of a head in clay copied in porcelain.

He turned, and his eyes met those of Brydges. Darting a keen glance at the young man, in which one could almost have fancied there was an expression of defiance; he said, abruptly: "My daughter tells me you are from Mobile. How long have you lived there?"

"All my life."

"Have you relatives of your name in Savannah?"

"No."

Brydges was a little annoyed at this peremptory interrogatory, and so answered very curtly. He did not feel inclined to say that his father had formerly resided in Savannah, but had married and settled in Mobile.

The intense expression vanished at once from the face of Des Ponts. He smiled cheerily, with a flash of splendid white teeth, and said:

"Pardon my summary questions—a relic of my bad lawyer habits. An accidental resemblance, doubtless. Colonel Marshall, Mr. Brydges is a proof of what I have so often told you, that you will find the pure blonde Saxon type oftener in the South than the North."

This remark induced an ethnological controversy between the two gentlemen, which lasted until dinner, and Brydges forgot the explanation he had intended to make.

In the evening Mrs. Marshall said, "Now, Mimi, you must sing for me. I get no music except in these flood times."

"I will sing you something entirely new, by a composer whose name I never heard before—a Mr. Boote, who lives in Florence. A friend of mine traveling in Italy copied and sent it to me. It takes hold of me wonderfully."

She sang, in a rich, powerful, vibrating contralto, a wild lawless, but singularly thrilling air, to the words of Kingsley's "Sands o'Dee."

There came over Brydges, as she sang, that sense of mysterious recognition which all have sometimes felt, when every word and gesture falls inevitably into its place, as if we had known and foreseen it all for a thousand years. The other persons in the room became as shadows. He knew the song would cease in a moment, and there would be shadowy words of applause from those outside spectres. But, while the wild, sobbing music lasted, he and she were alone in the world of sensuous melody. Every touch of her fingers on the pearl and ebony keys fell on his heart, and the song they waked was, "She is mine, and no other's. I love her. I have loved her forever."

The song ended, and the spell was broken. At Cade's request

Miss Des Ponts sang that brilliant serenade of Gounod's to Victor Hugo's delicious words, "*Chantez, riez, dormez.*" But Brydges only wondered at his ecstacy of a moment before. He looked with critical appreciation at the singer, and saw a superb young girl, as lovely as youth and beauty could make her, singing a showy song in an effective way. But, as if revenging himself for his momentary lapse from self-possession, he thought: "A very pretty girl—a little too *prononcée*—not infrequently slangy—needs a year or two of better society than she can find in Thebes."

The next day Miss Des Ponts started for a gallop on the Carthage road, attended by her two cavaliers. Cade deserted very soon, riding off to visit the Colonel's farm, north of the town. "It is a remarkably porous soil," said Cade. "Absorbs everything you put on it, and leaves no trace. Mimi, I hold you responsible for Mr. Brydges."

They rode an hour or two through the thick timber and the sunny lanes, and returned excellent friends. There was no resisting the charm of Marie's directness and sincerity. Her character had something manly in its frank, fearless honesty.

He was surprised at her unaffected sense of her own shortcomings. "I suspect it is not best for me to live as I do. Mamma died when I was a child, and I have grown up lawlessly with Victor. There—a new impropriety! I have always called him by his first name; instead of correcting me, he laughed and kissed me. So then I talked slang, till I am afraid I sometimes trip that way now, when I am old enough to know better. I read his books and his newspapers, and had no other education until Mrs. Marshall positively dragooned him into letting me go to school. I staid two years at the Visitation in St. Louis, and learned some music; then ran away and came back to him, and found him, I am sure, ten years older by the separation. I will not leave him again. And yet I know we ought not to live in that *triste* little town. I have so often begged him him to go South, or to Congress, or somewhere. With his great talents and influence he could do everything in politics. But he detests the very name. I believe he cares for nothing but me."

These words were uttered with an intonation indescribably sweet and winning. The great brown eyes were softened with unshed tears. But before Brydges could speak she struck her horse a smart blow with the riding whip, and they went dashing homeward, accompanied by a cloud of dust and the yelping of scandalized curs.

The days passed on pleasantly enough with walking and riding and making visits in Moscow, where Marie knew everybody and was universally admired. Mr. Des Ponts went every morning to Thebes, and passed an hour or two in his skiff, going from window to window of those acquaintances who had valorously remained in their upper rooms, lulled nightly to sleep by the rushing of waters under their floors. Day after day Mr. Brydges said, resolutely, "I go to-morrow." But the weather was finer than he had ever seen, the skies bluer than ever had shone, and the Marshalls were the pleasantest hosts he ever had met. So he lingered, and still was traveling always into the borders of the Enchanted Land, which is as old as nature, yet newer and fresher and stranger than anything on earth to each young heart that finds it. He never asked himself how far he should go. The path was smooth and enticing, the air subtle and fine. Continually just beyond there was a bank of rare blossoms, a splendor of sunlight on the emerald lawns. He would go that far, and then? All the while the shuttle of Fate was flying swiftly about him, and weaving into the web of his life a richness and brilliancy it had never known.

One evening he and Miss Des Ponts were sitting alone on the veranda. They had been talking for an hour. The conversation was of the river, of the news, of books; at first animated, then languid, till it dropped into an embarrassed silence. Clarence had given himself utterly up to the delight of his eyes. Her delicate profile was defined against the clear dark sky of the west. The light of the young May moon lay on her rippled hair. She seemed in the faint glimmer almost too lovely to be real. As the young man gazed at her he forgot that she was talking, and even answered her questions at random. Surprised and perplexed, she ceased speaking.

(TO BE CONTINUED.)

### JUST AS OF OLD.

Just as of old! The world rolls on and on;
The day dies into night—night into dawn—
Dawn into dusk—through centuries untold,
Just as of old.

Time loiters not. The turbid stream still flows,
Its brink or white with blossoms or with snows;
Its tide or warm with spring or winter cold,
Just as of old.

Lo, where is the beginning, where the end
Of this perplexing skein of life, my friend?
God answers with a silence of pure gold,
Just as of old.

—*James W. Riley.*

